# Atomistic Assessment of Solute-Solute Interactions during Grain Boundary Segregation

**DOI:** 10.3390/nano11092360

**Published:** 2021-09-11

**Authors:** Thomas P. Matson, Christopher A. Schuh

**Affiliations:** Department of Materials Science and Engineering, Massachusetts Institute of Technology, 77 Massachusetts Avenue, Cambridge, MA 02139, USA; tmatson@mit.edu

**Keywords:** grain boundary, segregation, atomistic modeling, solute interactions

## Abstract

Grain boundary solute segregation is becoming increasingly common as a means of stabilizing nanocrystalline alloys. Thermodynamic models for grain boundary segregation have recently revealed the need for spectral information, i.e., the full distribution of environments available at the grain boundary during segregation, in order to capture the essential physics of the problem for complex systems like nanocrystalline materials. However, there has been only one proposed method of extending spectral segregation models beyond the dilute limit, and it is based on simple, fitted parameters that are not atomistically informed. In this work, we present a physically motived atomistic method to measure the full distribution of solute-solute interaction energies at the grain boundaries in a polycrystalline environment. We then cast the results into a simple thermodynamic model, analyze the Al(Mg) system as a case study, and demonstrate strong agreement with physically rigorous hybrid Monte Carlo/molecular statics simulations. This approach provides a means of rapidly measuring key interactions for non-dilute grain boundary segregation for any system with an interatomic potential.

## 1. Introduction

Nanocrystalline metals exhibit a wide range of useful properties that often exceed what is achievable at the microscale [1,2,3,4,5,6,7,8,9,10]. However, they are often in unstable, nonequilibrium states due to a high concentration of grain boundaries (GBs) that contribute to the free energy of the system and create an increasingly large driving force for grain growth at the nanoscale [11,12,13,14]. Alloying can provide a means of thermodynamically stabilizing the nanocrystalline state by lowering the grain boundary energy via grain boundary solute segregation [11,15,16,17,18,19,20,21,22,23,24,25,26,27,28,29,30]. This thermodynamic approach has been gaining increased attention in recent years compared to kinetic methods of stabilization [31,32,33,34,35,36], due to its reliability and relatively simple design space, which requires only thermodynamic knowledge of the alloy system. 

Prior work in this area has focused on the development of models that can predict the segregated state of alloy systems. For example, there are a number of isotherm models that predict *GB* solute concentrations [37], including those of McLean [38], Fowler-Guggenheim [39], Guttman [40], and Wynblatt and Chatain [41]. In more recent years, this approach has been extended to specifically consider the nanocrystalline state with regular solution, lattice Monte Carlo, and phase-field models [24,26,27,28,42]. However, a major shortcoming of most all such models is their use of a single segregation energy to characterize the entire grain boundary network, which in reality has a complex diversity of segregation sites. The shortcomings of this assumption were recently analyzed and corrected by Wagih and Schuh [43]. Taking inspiration from the works of White and Stein [44] and Kirchheim [45,46], they used a spectral McLean-type isotherm, in which each atomic grain boundary site has its own dilute limit segregation energy, and calibrated it directly to atomistic results on nanocrystalline structures. 

Wagih and Schuh demonstrated that the spectral approach achieves significantly better agreement with the results of full atomistic simulations on Al-Mg polycrystals. More recently, they addressed the issue of solute-solute interactions [47], a necessary consideration away from the dilute limit, when solutes begin to interact in the GBs, locally affecting *GB* segregation there. In the spectral model, there is a wide range of such interactions, and rather than treat them all, Wagih and Schuh showed that the addition of a single, fitted interaction energy (assumed relevant to all sites) could account for non-dilute interactions in an average sense, with good agreement to the overall segregated solute concentrations [47]. However, because the parameter calculated by Wagih and Schuh was simply fitted to the results of atomistic simulations, it is not derived from atomistic-level physics directly. As a result, it is not generalizable without expensive computations on each individual alloy. 

The focus of this work is therefore to seek a physically motivated atomistic method to assess solute interactions during grain boundary segregation, in a way that acknowledges the wide diversity of sites and can be easily incorporated into existing spectral isotherm models. For the Al-Mg system, we show how atomistic simulations can be used to measure the full spectrum of solute interactions over the full spectrum of segregation sites in a polycrystal. The results of such simulations lead to a simple hypothesized general form for *GB* solute interactions for future modeling efforts. 

## 2. Thermodynamics of Grain Boundary Segregation

### 2.1. Free Energy vs. Enthalpy of Segregation

A rigorous thermodynamic treatment of *GB* segregation must consider the Gibbs free energy of segregation [48], $\Delta G_{seg}$. The segregation free energy includes not only the enthalpic contribution considered above, $\Delta E_{seg}$, but also a work term, −PΔV, where P is the pressure and ΔV is the volume change, as well as a vibrational entropy term, $-T\Delta S_{seg}^{vib}$ [49], such that the free energy of segregation is given as:

(1) $$\begin{array}{c} \Delta G_{seg}=\Delta E_{seg}-P\Delta V-T\Delta S_{seg}^{vib}. \end{array}$$

However, the vibrational entropy component of *GB* segregation is generally not well understood, and can be neglected at reasonably low temperatures, as we do here. Furthermore, PΔV is generally negligible in solids [50], and is neglected here. Self-consistency is achieved by using only enthalpic measurements of the segregated states at 0 K via conjugate gradient minimization. Thus, even though configurational space is sampled at finite temperature during the following simulations, vibrational contributions are consistently neglected, and we can assume that $\Delta G_{seg}\approx \Delta E_{seg}$ in the isotherms presented below. 

### 2.2. Classical Segregation Models 

The first isotherm for grain boundary segregation was proposed by McLean [38], in which the segregation energy is taken to be a single average parameter, $\Delta {\bar{E}}^{seg}$, given as the difference in energy of the full system when a solute, B, occupies a grain boundary site, $E_{GB}^{B}$, vis-à-vis a bulk site, $E_{c}^{B}$:

(2) $$\begin{array}{c} \Delta {\bar{E}}^{seg}=E_{GB}^{B}-E_{c}^{B}\; \end{array}$$

This approach assumes that the segregation energy, $\Delta {\bar{E}}^{seg}$, is independent of grain boundary character (or the site occupied by the solute), solute concentration, and temperature (T), resulting in McLean’s isotherm [38]:

(3) $$\frac{{\bar{X}}^{GB}}{1-{\bar{X}}^{GB}}=\frac{X^{c}}{1-X^{c}}\exp\left(-\frac{\Delta {\bar{E}}^{seg}}{kT}\right)$$

where ${\bar{X}}^{GB}$ is the average solute concentration in the *GB*, $X^{c}$ is the concentration in the bulk, and k is Boltzmann’s constant. 

To extend this treatment beyond the dilute limit, Fowler and Guggenheim accounted for concentration dependence of the segregation energy via the addition of a single interaction parameter based on a heat of mixing in the *GB*, $\Omega^{GB}$ [39]:

(4) $$\frac{{\bar{X}}^{GB}}{1-{\bar{X}}^{GB}}=\frac{X^{c}}{1-X^{c}}\exp\left(-\frac{\Delta {\bar{E}}^{seg}+2\Omega^{GB}{\bar{X}}^{GB}}{kT}\right)$$

which assumes that solute interactions in the bulk are negligible, due primarily to the assumption of relatively large, dilute grains, and thus relatively constant, dilute values of $X^{c}\approx X^{tot}$, where $X^{tot}$ is the total system solute concentration. This assumption can be corrected with the addition of a term that includes the bulk heat of mixing, $\Omega^{c}$ [51]. This term appears consistently in more recent models that explicitly consider the nanocrystalline grain sizes [24,26,27,28,42], and when combined with the mixture rule, where $X^{tot}$ is fixed and $X^{c}$ and ${\bar{X}}^{GB}$ can vary dependently as [52]:

(5) $$\begin{array}{c} X^{tot}=\left(1-f^{GB}\right)X^{c}+f^{GB}{\bar{X}}^{GB}, \end{array}$$

results in the complete isotherm for nanocrystalline alloys:

(6) $$\begin{array}{c} X^{tot}=\left(1-f^{GB}\right)X^{c}+f^{GB}\;{\left[1-\frac{1-X^{c}}{X^{c}}\exp\left(\frac{\Delta {\bar{E}}^{seg}-2\Omega^{GB}{\bar{X}}^{GB}+2\Omega^{c}X^{c}}{kT}\right)\right]}^{-1} \end{array}$$

where $f^{GB}$ is the volume fraction of the grain boundary, and is typically related to the grain size, d, and grain boundary thickness, t, by the equation:

(7) $$\begin{array}{c} f^{GB}=1-{\left(\frac{d-t}{d}\right)}^{3}. \end{array}$$

Assuming only nearest-neighbor contributions for solvent A and solute B, the heat of mixing can be represented as:

(8) $$\begin{array}{c} \Omega^{s}=\frac{1}{2}z^{s}w^{s}=\frac{1}{2}z^{s}\left(E_{A-B}^{s}-\frac{E_{A-A}^{s}+E_{B-B}^{s}}{2}\right) \end{array}$$

where s refers to either the *GB* or the bulk, z is the atomic coordination, and $E_{A-B}^{s}$, $E_{A-A}^{s}$, and $E_{B-B}^{s}$ are the bond energies of A-B, A-A, and B-B bonds, respectively. 

### 2.3. Spectral Segregation Models 

Following the density of sites approach introduced by White and Stein [44] and Kirchheim [45,46], Wagih and Schuh developed a spectral model for grain boundary segregation, which assumes that each atomic grain boundary site has its own dilute limit segregation energy. Assuming a McLean-type contribution from each site type i with dilute limit segregation energy $\Delta E_{i}^{seg}$, and accounting for the mixture rule of Equation (5), Wagih and Schuh’s spectral isotherm is given as an integral over segregation energies [43]:

(9) $$\begin{array}{c} X^{tot}=\left(1-f^{GB}\right)X^{c}+f^{GB}\int_{-\infty }^{\infty }F_{i}^{GB}{\left[1+\frac{1-\;X^{c}}{X^{c}}\exp\left(\frac{\Delta E_{i}^{seg}}{kT}\right)\right]}^{-1}d\left(\Delta E_{i}^{seg}\right) \end{array}$$

where $F_{i}^{GB}$ is the density of sites of type i, and was shown by Wagih and Schuh to follow a roughly skew-normal distribution for general polycrystals:

(10) $$\begin{array}{c} F_{i}^{GB}=\frac{1}{\sqrt{2\pi }\sigma }\exp\left[-\frac{{\left(\Delta E_{i}^{seg}-\mu \right)}^{2}}{2\sigma^{2}}\right]\mathrm{erfc}\left[-\frac{\alpha \left(\Delta E_{i}^{seg}-\mu \right)}{\sqrt{2}\sigma }\right] \end{array}$$

where α, μ, and σ are the fitted shape, location, and breadth of the dilute limit segregation energy distribution, respectively. The values of these parameters for several hundred binary alloys have been presented in reference [53].

Following from Equation (4), this spectral isotherm can be adapted to account for solute interactions in the grain boundary with a single Fowler-type interaction parameter:

(11) $$\begin{array}{c} X^{tot}=\left(1-f^{GB}\right)X^{c}+f^{GB}\;\int_{-\infty }^{\infty }F_{i}^{GB}{\left[1-\;\frac{1-X^{c}}{X^{c}}\exp\left(\frac{\Delta E_{i}^{seg}-2\Omega^{GB}{\bar{X}}^{GB}}{kT}\right)\right]}^{-1}d\left(\Delta E_{i}^{seg}\right). \end{array}$$

Wagih and Schuh showed that for the Al-Mg system, the grains remain dilute even as the *GB* segregation raises the concentration locally at the boundary, leading to a significant effect via $\Omega^{GB}$; thus, a single fitted value of $\Omega^{GB}$ provided a reasonably accurate description of full atomistic simulations beyond the dilute limit [47]. For other nanocrystalline alloys, the bulk concentration may vary more significantly, so for completeness it is appropriate to use both *GB* and bulk contributions to the interactions, as in Equation (6). Thus, the isotherm of Equation (9) can be extended to account for non-dilute interaction as follows:

(12) $$\begin{array}{c} X^{tot}=\left(1-f^{GB}\right)X^{c}+f^{GB}\;\int_{-\infty }^{\infty }F_{i}^{GB}{\left[1-\;\frac{1-X^{c}}{X^{c}}\exp\left(\frac{\Delta E_{i}^{seg}-2{\bar{\Omega }}^{GB}{\bar{X}}^{GB}+2\Omega^{c}X^{c}}{kT}\right)\right]}^{-1}d\left(\Delta E_{i}^{seg}\right) \end{array}$$

where ${\bar{\Omega }}^{GB}$ and $\Omega^{c}$ are the average heat of mixing parameters of the grain boundary and bulk, respectively. The overbar on the former term is introduced to acknowledge that this $\Omega^{GB}$ is no longer formally a single parameter in the spectral model, as there are many sites with unique behaviors. Assessing the average value over many sites from atomistic information will be the major focus of our efforts below.

## 3. Atomistic Simulation Methods

### 3.1. Production of Pure Al Polycrystal 

**Figure 1:**
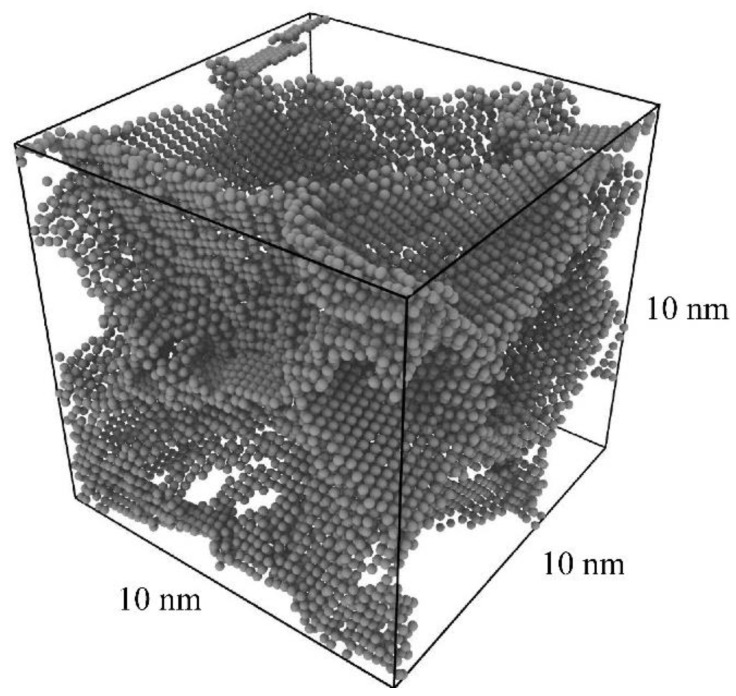
Visualization of the grain boundary network of the pure Al polycrystal after relaxation and annealing, with dimensions of (10 nm)^3^, 10 randomly oriented grains of average diameter 6 nm, and 60,367 total atoms.

A cubic polycrystal of pure aluminum was produced, with dimensions of (10 nm)^3^, 60,367 total atoms, and 10 grains of random orientation with an average diameter of 6 nm (Figure 1). The polycrystal was randomly initialized via Voronoi tessellation using the toolkit Atomsk (Version b0.11.1, University of Lille, Villeneuve d’Ascq, France) [54], followed by structural relaxation with conjugate gradient minimization. The polycrystal was then thermally annealed in an isothermal isobaric ensemble with a Nose-Hoover thermostat/barostat, at zero pressure and a temperature of 600 K for 0.5 ns. Finally, the polycrystal was cooled to 0 K over 0.25 ns, followed by a final conjugate gradient minimization. 

An image of the grain boundary network is shown in Figure 1, using polyhedral template matching to identify non-face-centered-cubic (non-FCC) regions in the Open Visualization Tool OVITO (Version 3.5.0, Darmstadt University of Technology, Darmstadt, Germany) [55]. All simulations here and in the remainder of this work were performed with the LAMMPS simulation package (Version 7Aug19, Sandia National Laboratories, Albuquerque, NM, USA) [56] and use the embedded atom method (EAM) potential by Mendelev for Al-Mg [57].

Here it should be noted that the (10 nm)^3^ polycrystal used in this work, at an average grain size of 6 nm, is significantly smaller than the (15 nm)^3^ and (36 nm)^3^ polycrystals used by Wagih and Schuh previously [43,47], with grain sizes of 9 and 12 nm, respectively. However, preliminary work in analyzing the grain size dependence of the segregation energy distribution indicates that changes in the distribution with respect to grain size are due primarily to the increased presence of triple junctions and quadruple nodes at smaller grain sizes. While this effect is non-negligible, for most alloys, including Al-Mg, the effective difference in segregation energy when decreasing the grain size from 12 nm to 6 nm is of at least an order of magnitude less than the effective segregation energy itself. 

### 3.2. Dilute Limit Segregation Energy Distributions

**Figure 2:**
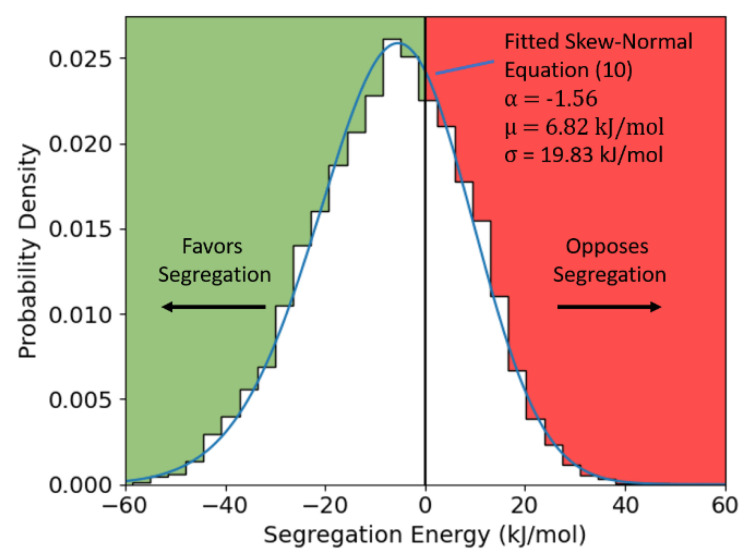
Dilute limit segregation energy distribution for Al-Mg, calculated from the (10 nm)^3^ polycrystal, with a fitted skew-normal distribution overlaid.

The Al-Mg system studied in this work was chosen for the strong agreement between its available interatomic potential [57] and density functional theory [58] when calculating segregation energies, and because it has been previously used for spectral *GB* segregation analysis [47]. To compute the dilute limit segregation energy distribution of the Al polycrystal, we follow the procedure of Wagih and Schuh [43]. We compute the energy difference between the fully relaxed polycrystal with a single solute atom, B, at *GB* site i, $E_{GB,\;\;i}^{B}$, or at a bulk site in the center of the largest grain, $E_{c}^{B}$:

(13) $$\begin{array}{c} \Delta {\bar{E}}_{i}^{seg}=E_{GB,\;\;i}^{B}-E_{c}^{B} \end{array}$$

and systematically test every site lacking FCC coordination. The resulting discrete distribution for Al-Mg is thus shown in Figure 2, with a skew-normal function fitted to Equation (10) overlaid. The distribution calculated here is skew-left, spans from approximately −60 to 40 kJ/mol, and has a mean of −6.82 kJ/mol, all of which are in excellent agreement with the distribution calculated previously by Wagih and Schuh for a (36 nm)^3^ polycrystal [43]. 

**Figure 3:**
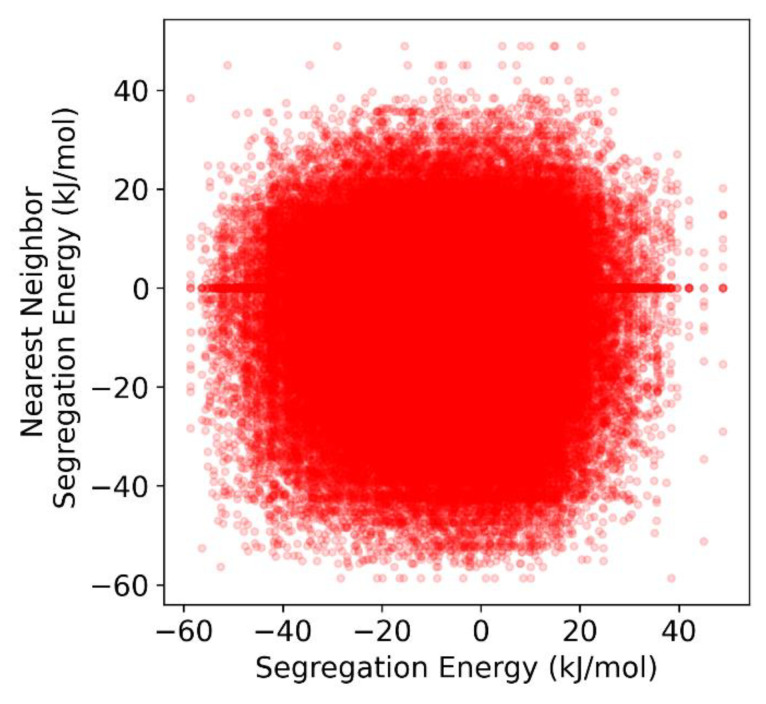
Correlation plot of the average segregation energy of the nearest neighbors of a given grain boundary site vs. the segregation energy of that site for Al-Mg in the (10 nm)^3^ polycrystal.

Because the isotherm models presented in Section 2 assume random mixing in the grain boundary in order to derive the linear interaction parameters that we are attempting to measure, it is necessary to demonstrate that random mixing is a reasonable assumption to make for the Al-Mg polycrystal used in this work. Wagih and Schuh have already shown using a two-point correlation function that, for random polycrystals with general grain boundaries, such as those used in this work, grain boundary sites of a given segregation energy are approximately randomly distributed along the grain boundary network in Al-Mg [43]. To demonstrate this in a simple manner, Figure 3 plots the relationship between the segregation energy of a given grain boundary site, and the average segregation energy of its nearest neighbors, identified using a Voronoi analysis. For this Al-Mg polycrystal, it is readily apparent that there is little to no correlation between a site’s segregation energy, and those of its nearest neighbors. This, in combination with the random distribution of segregation energies along the *GB* network, indicates that random mixing in the grain boundary is a reasonable approximation from which to assess solute interactions in the *GB* for this system. 

It should be stressed, however, that such a random distribution of solutes is achieved generally only in the case of mild solute-solute interactions at the grain boundary. This condition occurs when the segregation energy dominates over the interaction energy—for segregation energy distributions with particularly large negative tails, and at concentrations low enough to access primarily those *GB* sites—or at temperatures high enough to thermalize the interactions (but not *GB* segregation itself) and achieve some semblance of random mixing. If the interactions are stronger, random mixing may not occur at relevant temperatures. For example, we have found that in systems with strong attractive interactions the solutes readily cluster upon *GB* segregation, as a prelude to outright phase separation. For the present analysis, the competition between second phase formation and *GB* segregation is explicitly not of interest (although it has been addressed in prior work in the dilute limit [59] and we will address it in our future work beyond the dilute limit). Future work should address in more detail how a given system may be explored to achieve these conditions; for the moment we can proceed with confidence that the Al-Mg system is a viable case study for the proposed model.

### 3.3. The True Equilibrium Segregation State: Hybrid MC/MS

To evaluate the predictions of the procedure proposed in this work, it is necessary to obtain the equilibrated segregation state of our Al-Mg polycrystal with finite solute content. This is done using a standard Monte Carlo (MC) procedure at a finite temperature to sample configurational space, in combination with molecular statics relaxations [17,60,61,62,63,64,65,66,67]. The Al polycrystal shown in Figure 1 was randomly populated with Mg solute, at concentrations of $X_{tot}$ up to 10 percent. One step in the hybrid MC/MS procedure, referred to as one MC step, was conducted as a series of micro-MC steps at finite temperature, followed by a full-system relaxation at 0 K and constant pressure. Each micro-MC step consisted of a Monte-Carlo swap, attempted with a probability given by the metropolis criterion at 600 K, using the EAM potential for all energy evaluations. 6000 micro-MC steps were attempted per MC step in the hybrid MC/MS procedure. 1000 to 2000 MC steps, scaling linearly with total solute concentration, were conducted to reach adequate convergence in both system energy and solute distribution. 

**Figure 4:**
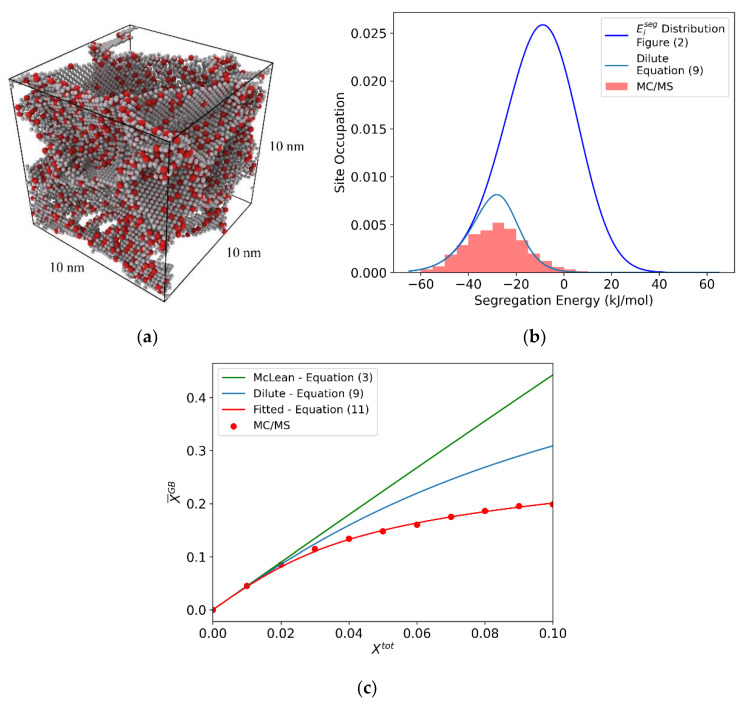
(**a**) Al-Mg polycrystal with 5% total solute, equilibrated with hybrid MC/MS at 600 K. (**b**) Segregation energy distribution with the equilibrium occupied distribution shown in red. Predicted occupied distribution is shown for the dilute case (Equation (9) (blue)). (**c**) For the (10 nm)^3^ Al-Mg polycrystal: McLean-style isotherm with effective segregation energy $\Delta {\bar{E}}_{eff}^{seg}$ = −26.5 (Equation (3) (green)), dilute limit spectral isotherm (Equation (9) (blue)), and polycrystal equilibrated via MC/MS, with a fitted linear interaction parameter $\Omega^{GB}$ = −22.86 kJ/mol (Equation (11) (red)).

The final state of the system after this process is taken as the true equilibrium segregation state, from which the final solute distribution is measured. An example equilibrated polycrystal of Al-Mg at $X_{tot}$ = 0.05 is shown in Figure 4a. The distribution of occupied sites is shown in red in Figure 4b, and resembles prior work on this system from Ref. [47]. These occupation distributions represent the true equilibrium segregation state, which we intend to understand in terms of Equation (12).

The resulting equilibrium grain boundary solute concentration, $X^{GB}$, is plotted as a function of $X^{tot}$, shown as red points in Figure 4c. In the work of Wagih and Schuh [47], Equation (11) was simply fitted to simulation results such as these, treating the solute interaction parameter(s) as unknown constants. Following this same approach here, as shown in Figure 4c, results in a value of $\Omega^{GB}$ = −22.86 kJ/mol. For comparison, a McLean-style isotherm is plotted in green, using an effective segregation energy, $\Delta {\bar{E}}_{eff}^{seg}$ = −26.5 kJ/mol, fitted from Equation (3) in the dilute limit. Equation (9), which includes the effect of the segregation energy spectrum in the dilute limit, is also shown in blue.

This result, while physically motivated by the work of Fowler and Guggenheim [39], is ultimately a fitted parameter that is not derived from atomistic-level physics directly, and requires relatively expensive simulations to compute. Additionally, the use of a single interaction parameter does not explicitly separate the interaction contributions from the bulk and grain boundary. Our goal here is to instead seek a direct atomistic assessment of those parameters, and success will be measured by our ability to reproduce the true segregation state in Figure 4b,c.

### 3.4. Grain Boundary Heat of Mixing Distributions

Use of the isotherm given in Equation (12) requires knowledge of an average heat of mixing parameter for both the bulk and grain boundary. We are not aware of any prior measurement of the full distribution of the heat of mixing across all grain boundary sites, so we proceed to make one here. To separate the contributions of coordination and bond energy distributions in the grain boundary, we calculate the per-bond parameter $w^{GB}$, as given in Equation (8), in addition to the coordination of each *GB* site.

**Figure 5:**
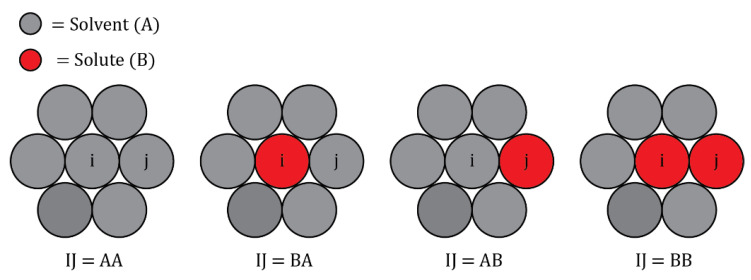
Example 2d atomic configurations used to calculate the per-bond parameter $w_{ij}^{GB}$ for bond i-j, by measuring the per-atom energy of atom *I* in the fully relaxed polycrystal, $E_{ij,\;IJ}^{GB}$, where atoms *I* and *J* can be either solvent A or solute B.

The coordination of each grain boundary site is calculated via Voronoi analysis in the OVITO visualization tool. $w^{GB}$ is then extracted for each nearest neighbor bond of each grain boundary site, including *GB*-bulk bonds, in the following manner. For a given *GB* site i and nearest neighbor site j, the per-atom energy of atom I in the fully relaxed polycrystal, $E_{ij,\;IJ}^{GB}$, is calculated for each of the example 2D configurations shown in Figure 5, where atoms I and J can be occupied by either a solvent atom A or solute atom B, and the energy of each configuration is given as:

(14) $$\begin{array}{c} E_{ij,\;\;xy}^{GB}=\frac{1}{2}[(z_{i}^{GB}-1)E_{y-x}^{GB}+E_{ij,\;x-y}^{GB}] \end{array}$$

where *x* and *y* can be either solute A or solvent B in the four possible permutations shown in Figure 5, and the per-bond parameter $w_{ij}^{GB}$ for bond i-j can be calculated as:

(15) $$\begin{array}{c} w_{ij}^{GB}=\left(E_{ij,A-B}^{GB}-\frac{E_{ij,AA}^{GB}+E_{ij,BB}^{GB}}{2}\right)=E_{ij,\;BA}^{GB}-E_{ij,\;BB}^{GB}+E_{ij,\;AB}^{GB}-E_{ij,\;\;AA}^{GB}. \end{array}$$

The parameter $w_{ij}^{GB}$ can then be averaged over each nearest neighbor for a given *GB* site i to obtain an average per-site parameter $w_{i}^{GB}$. This value can in turn be combined with the atomic coordination of the site to obtain the per-site heat of mixing parameter, $\Omega_{i}^{GB}$, and thus the full heat of mixing distribution of the grain boundary. Here, it should be noted that the heat of mixing parameters calculated effectively assume the structure of the pure solvent A—in either the grain boundary or bulk, respectively—as the reference state for both components A and B.

**Figure 6:**
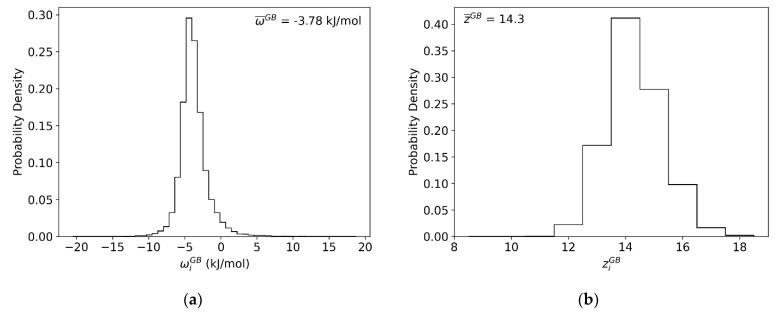

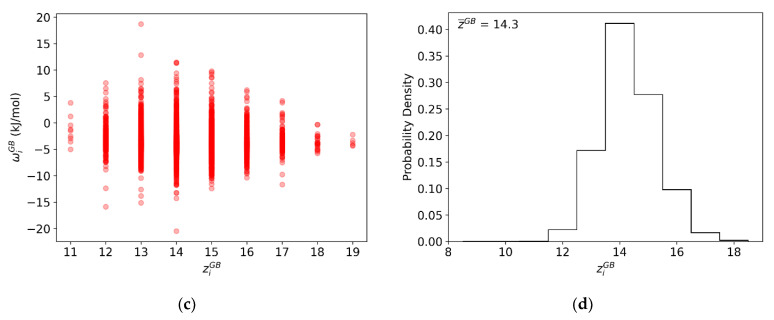
For every *GB* site in the (10 nm)^3^ Al-Mg polycrystal: (**a**) Atomic coordination of every *GB* site. (**b**) Correlation plot of atomic coordination and per-site parameter $w_{i}^{GB}$. (**c**) Average per-site parameter $w_{i}^{GB}$. (**d**) Average per-site heat of mixing parameter $\Omega_{i}^{GB}$.

Following this procedure for a bulk site in the interior of a fully relaxed 16 × 16 × 16 supercell of FCC Al, values for the grain interior of $z^{c}=12$, $w^{c}=$ −4.72 kJ/mol, and $\Omega^{c}=$ −28.32 kJ/mol were obtained. Then, following this procedure for the GBs, we achieve the distribution shown in Figure 6a. This per-site parameter exhibits a roughly skew-normal distribution, similar to the segregation energy spectrum itself, with an average value of ${\bar{w}}^{GB}=$ −3.78 kJ/mol. We note that this spectrum confirms our earlier observations about the modest nature of solute-solute interactions in Al-Mg, as it is far less energetic than the *GB* segregation spectrum itself (cf. Figure 2); this means that at low temperatures, thermal energy is enough to randomize the solute-solute interactions in the GBs but not to desegregate them, achieving exactly the random mixing conditions required to evaluate solute interactions.

To directly compare the measured per-site parameter $w_{i}^{GB}$ with the fitted parameter $\Omega^{GB}$, we must also account for the atomic coordination $z_{i}^{GB}$ of each *GB* site, as per Equation (8). However, $w_{i}^{GB}$ and $z_{i}^{GB}$ are not necessarily independent. Thus, to explicitly separate the contributions due to coordination and bond energy distributions, the atomic coordination distribution of the grain boundary, $z_{i}^{GB}$, was also measured and is shown in Figure 6b. When plotting $w_{i}^{GB}$ as a function of $z_{i}^{GB}$, as shown in Figure 6c, it is readily apparent that the spread of $w_{i}^{GB}$ varies significantly with atomic coordination. However, there is very little overall correlation between the two, so their rigorous site-wise combination to produce a spectrum as in Figure 6d, followed by averaging, produces much the same result as first averaging each distribution and then using Equation (8) subsequently. This analysis gives an average heat of mixing parameter for the *GB* regions as ${\bar{\Omega }}^{GB}=$ −27.10 kJ/mol.

## 4. Discussion

**Figure 7:**
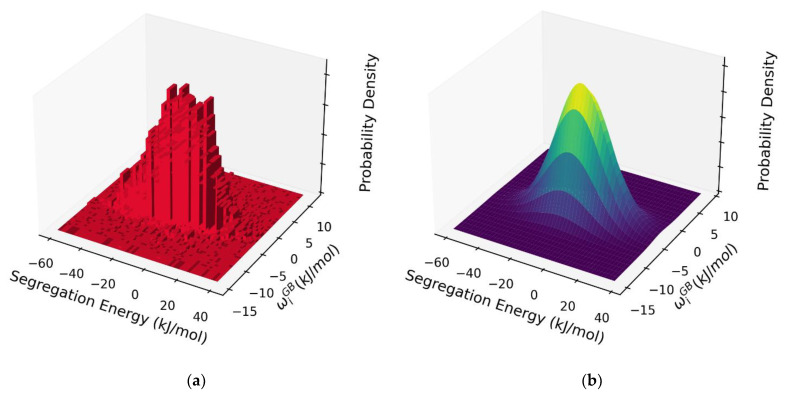
(**a**) 2D histogram of the dilute limit segregation energy and per-site interaction parameter $w_{i}^{GB}$, exhibiting a bivariate skew-normal distribution. (**b**) Bivariate normal distribution fitted to the data depicted in Figure 7a.

The results in Figure 6 represent what we believe to be the first atomistic measurement of the full spectrum of solute-solute interaction effects during *GB* segregation in a polycrystal. As such, they permit a very detailed level of analysis of the *GB* segregation state beyond the dilute limit. For example, in the spirit of exhaustive rigor, we might consider an isotherm analysis on the basis of both the spectrum of segregation energies and the spectrum of solute interactions across the *GB*, combined together in a self-consistent probabilistic model. This is explored in Figure 7a, where the per-site dilute limit segregation energy and interaction parameter are cross-compared, and together apparently constitute a single 2D distribution function with a single central peak. 

Such a distribution could be modeled by, e.g., a bivariate normal (or skew-normal) distribution [68]. Equation (9) might therefore be modified to include an integral over the joint probability density of the segregation and interaction energies. The skewness is small in the present case, so a bivariate normal distribution is appropriate, and has the following form:

(16) $$\begin{array}{c} F_{ij}^{GB}=\frac{1}{\sqrt{{\left(2\pi \right)}^{2}\left|\Sigma \right|}}\exp\left[-\frac{1}{2}{\left(x-µ\right)}^{T}\Sigma^{-1}\left(x-µ\right)\right] \end{array}$$

where $F_{ij}^{GB}$ varies with the vector quantities x and µ, where x contains the segregation and interaction energies and µ their means, and Σ is their covariance matrix. For Al-Mg, we find the bivariate normal parameters to be $µ=\begin{array}{cc} {}[\Delta {\bar{E}}^{seg}, & {\bar{w}}^{GB}] \end{array}$, where $w{\bar{E}}^{seg}$ = −7.10 kJ/mol is the mean segregation energy and ${\bar{\Delta }}^{GB}$ = −3.78 kJ/mol is the mean interaction energy, with a covariance matrix given by:

$$\Sigma =\left[\;\begin{array}{cc} 244.04 & 4.76\\ 4.76 & 3.86 \end{array}\;\right]\;\mathrm{kJ}/\mathrm{mol}.\;$$

Performing an integration over both the segregation energy and interaction energy produces the following isotherm:

(17) $$\begin{array}{c} \begin{array}{c} X^{tot}=\left(1-f^{GB}\right)X^{c}\\ +f^{GB}\;\int_{-\infty }^{\infty }\int_{-\infty }^{\infty }F_{ij}^{GB}{\left[1-\;\frac{1-X^{c}}{X^{c}}\exp\left(\frac{\Delta E_{i}^{seg}-2{\bar{\Omega }}_{j}^{GB}{\bar{X}}^{GB}+2\Omega^{c}}{kT}\right)\right]}^{-1}d\left({\bar{\Omega }}_{j}^{GB}\right)d\left(\Delta E_{i}^{seg}\right). \end{array} \end{array}$$

**Figure 8:**
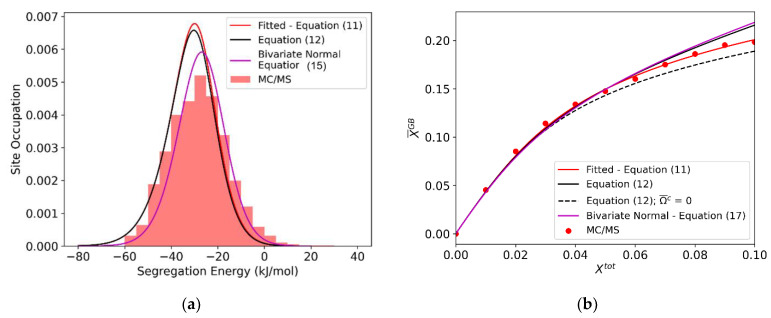
(**a**) For the (10 nm)^3^ Al-Mg polycrystal: isotherm for the polycrystal equilibrated via MC/MS at 600 K, with a fitted linear interaction parameter $\Omega^{GB}$ = −22.86 kJ/mol (Equation (11) (red)), spectral isotherm with the average bulk interaction parameter $\Omega^{c}=$ −28.32 kJ/mol and average grain boundary interaction parameter ${\bar{\Omega }}^{GB}=$ −27.10 kJ/mol (Equation (12) (solid black)), and spectral isotherm with fitted bivariate normal distribution (Equation (17) (magenta)). (**b**) Equilibrium occupied distribution, with predicted occupation distributions using: a fitted linear interaction parameter $\Omega^{GB}$ = −22.86 kJ/mol (Equation (11) (red)), average interaction parameters $\Omega^{c}=$ −28.32 kJ/mol and ${\bar{\Omega }}^{GB}=$ −27.10 kJ/mol (Equation (12) (black)) and the full fitted bivariate normal distribution (Equation (17) (magenta)).

Equation (17) can be readily solved numerically, and the resulting occupation distribution and isotherm are shown in magenta in Figure 8 for Al-Mg. When this fully atomistic solution is compared with the single-parameter Fowler-like fit in the details of the atomic site distributions (Figure 8a), it is clear that the full bivariate distribution more accurately captures the distribution at equilibrium. It also credibly reproduces the trend of the isotherm in Figure 8b with no fitting parameters. Interestingly, though, the conformity in Figure 8b is not better than can be achieved with direct fitting. Thus, even though the full bivariate distribution approach may be more rigorous, it may not dramatically improve predictive power over a simple linear interaction term, if one is concerned only with the average *GB* solute concentration and does not care about the details of site occupation. Since the full bivariate spectrum approach adds significantly more computational complexity, an atomistically-informed single parameter model may be a preferred solution. Introducing the directly atomistically measured average values of ${\bar{\Omega }}^{GB}$ and $\Omega^{c}$ into Equation (12) achieves the results shown by black lines in Figure 8; the result is a reasonable compromise between accuracy and speed.

One additional result is provided by the dashed black line in Figure 8b. This is the prediction of Equation (12) if only the solute-solute interactions in the *GB* are considered, and not the bulk interactions. As anticipated, in Al-Mg, this effect is relatively small, but not insignificant, especially at higher concentrations, indicating the need to account for both the grain boundary and bulk contributions in the general case. 

The above analysis shows that the general approach of using a Fowler-like composition-dependent correction to the spectral model, as proposed by Wagih and Schuh, is indeed an excellent compromise between simplicity and accuracy to capture *GB* segregation beyond the dilute limit. However, the manner of its use proposed by those authors is computationally cumbersome: in order to rigorously compute the true segregation state in Figure 8 by MC/MS and then fit the interaction parameter takes on the order of 200 h of compute time on a system using graphics processing unit (GPU) accelerated potential calculations with a Nvidia Quadro P4000 graphics card (NVIDIA, Santa Clara, CA, USA) and an Intel i7 4770 K processor (Intel, Santa Clara, CA, USA). In contrast, knowing that only a single average interaction value is needed, the present method based on direct atomistic sampling of solute-solute interactions becomes remarkably efficient. Rather than obtain the entire interaction spectrum as in Figure 6, we may instead take small samples to obtain just its mean. For the distribution shown in Figure 6d, the standard deviation of the distribution is $\sigma_{\Omega }^{GB}$ = 14.2 kJ/mol, and a sample size of n = 100 *GB* sites would be sufficient to reduce the standard error of the distribution mean to $\sigma_{\bar{\Omega }}^{GB}$ = 1.42 kJ/mol. These hundred computations would take about 1/100 the time of the MC/MS approach above. In future work we hope to apply this advance to rapidly screen solute-solute interactions for many alloys. 

## 5. Conclusions

Recent progress in accounting for the full spectrum of *GB* segregation sites has brought new clarity to the dilute limit situation but left the important topic of solute-solute interactions at higher concentrations in need of development. Here we have explored the natural extension of the spectral model for *GB* segregation by assessing a comparable distribution of solute-solute interaction energies. The method presented here has provided what is, to our knowledge, the first measurement of the full spectrum of solute-solute interaction energies at the GB. The spectrum of interaction energies follows a roughly skew-normal distribution for the Al-Mg system analyzed here, and when combined with the existing segregation energy distribution constitutes a full bivariate (skew-) normal distribution that describes the *GB* beyond the dilute limit. 

A full bivariate normal distribution of site and interaction energies provides an excellent prediction of the solute distribution at equilibrium, as validated against rigorous hybrid Monte Carlo/Molecular statics simulations, both on average and over the full spectrum of *GB* sites. Importantly, though, in the present case the interactions can be approximated by a scalar average over their full distribution and still achieve reasonable accuracy for many practical problems. This compromise is one that has the benefit of being fully atomistically informed, but less computationally intensive. This work thus paves the way to use simple, inexpensive atomistic measurement to predict solute interaction behavior during grain boundary segregation.
